# 

**Published:** 1885-11-20

**Authors:** 


### TABLE OF CONTENTS.

ORIGINAL AND SELECTED ARTICLES:

A Case of Myoma of the Cervix Uteri, by Virgil O. Hardon, M. D., Atlanta, Ga., 401. Icteroid Fever, by B. Frank Humphreys, M.D., Texas, 403 Urinary and Generative Organs, by John W. S. Gouley, M. D., Surgeon to Bell Hospital, New York, 408. Tyrotoxicon—Cheese Poison, Abstract of a paper by Prof. V. C. Vaughn, M.D , Ph. D., 412. On the Clinical Manifestations of Rotheln, or German Measles, by W. A. Edwards, M.D., Assistant Demonstrator of Clinical Medicine, University of Pennsylvania, 414. A Case of Poisoning by Hyoscyamin Treated by Morphia Hypodermically, by Robert Coltman, M. D., of Jenkintown, Pa , 416.

ABSTRACTS AND GLEANINGS:

Therapeutics of Jaborandi; The Hand, 417. Antiseptics; Treatment of Gonorrhœa in the Female, 418. Sajous on the Treatment of Hay Fever; Foreign Bodies in the Ear; Mistletoe a Parturifacient, 419. Unna's New Bougie Treatment of Chronic Gonorrhœa: Hypodermic Syringe; Removal of Tape-worm, 420. The Treatment of Typhoid Fever, 421. Poisoning by the Oil of Sassafras; For Stutterers, 423. Gelsemium Habit, 424. Progress in Medicine, 425. Hydronapthol, 426. To Prevent the Secretion of Milk in Still-born Cases, 427. New Method of Reducing Dislocations of the Hip; Tonsillitis; Potassium Bromide in Ovarian Menorrhagia, 428. The Last Episode of the Ferran Inoculations for Cholera: Veratrum as a Diaphoretic; The Microbe of Mumps; The Operation of Turning; Vomiting in Pregnancy, 429. An Antiseptic Ointment; Protective for Exposed Dental Nerves; For Urethral Discharges; To Keep Mosquitoes Away; A Cheap and Efficient Disinfectant, 430.

SCIENTIFIC ITEMS:

Another New Metal; Our Country; Experiments on the Decapitated, 431. A Paper Chimney; Electricity and Dust; Dyed Tomatoes, 432.

PRACTICAL NOTES AND FORMULÆ.

Ileo-colitis; Alopecia: To Relieve the Violent Pains of Lead Colic; Angostura Bitters; Pill for Syphilis, 433. For Sexual Debility; For Falling Hair; Villate's Mixture in Gleet; Freckle Wash; Hair Dye, 434.

EDITORIALS AND MISCELLANEOUS.

To Our Subscribers; Renewals; Soliloquy; The Difference ; Liberal Proposition, 435. The Record for 1886; Hope On, Hope Ever; Inoculation to Prevent Yellow Fever, 436. Prohibition in Atlanta, 437; Euthanasia; Price of Cocaine; Books and Pamphlets Received, 438. Receipted; Special Notices, 440.

